# Copy of a Letter from M. Percy, to the President of the First Class of the Institute, February, 1810, upon Some Platina Found at St. Domingo

**Published:** 1811-06

**Authors:** 


*Copy of a letter from* M. Percy, *to the President of the first Class of the Institute, February,* 1810, *upon some Platina found at St. Domingo.*

(Annales de Chimie.)

Sir,

I HAVE the honour to present the class with a specimen of Platina from St. Domingo, where this metal was not supposed to exist. I feel great pleasure in being able to offer it the first fruits of a work, which in its very commencement has been interrupted by the accidents of war.

Platina certainly exists in the Antilles. The present specimen was found in the river D'Iaky, at the foot of the mountain of Sibao. This river also carries along with it abundance of gold, especially after heavy rains. M. Dubizy, Surgeon-major, an eminent naturalist, and a man worthy of every confidence, returning from St. Domingo, procured some of the gold on the spot, and brought it to France. He separated it by means of the loadstone, from the sand which was mixed with it in considerable quantity.

Platina and gold are purchased in abundance, at very low prices, of the Spanish-Indian women, (femmes Indiennes-Espagnoles.)

The men are occupied in the chace, and do not engage in searching for and collecting the precious metals. St. Domingo is about 35 or 40 leagues from the mountain of Sibao, and when the communication was free, the French residing in that city made large profits in purchasing gold and platina.*

---

* When Columbus visited this island at the beginning of the 16th century, he obtained a plentiful supply of gold from the natives, who, before the arrival of the Spaniards, were accustomed to value this metal, which which they collected with great care. They contented themselves, however, with picking up small grains, which they readily found, and after having flattened them a little, made them into pendants for the nostrils. It is curious to remark the superstition which prevailed amongst these uninformed Indians; before a party proceeded on an expedition in search of gold, they prepared themselves by long fasting, and several days of *continence*, for the undertaking, which they declared would fail if they omitted this practice. Charlevoix informs us that Columbus wished to make his people observe a similar ceremony, and not to go to the mines *sans s'être auparavant approchés des Sacramens de Penitence et d'Encharistie.* They did not however approve of this pious restriction of their Chief, and remonstrated that the Church having only directed Confession and Communion, once a year, it was not for him to make new precepts. Besides they urged that the Spaniards having left their wives at home, were condemned to a much longer period of continence than that of the natives, and that with the scanty and bad diet to which they were reduced, they actually endured a continual and very rigorous fast. Editors.

